# Immunopeptidomics of colorectal cancer organoids reveals a sparse HLA class I neoantigen landscape and no increase in neoantigens with interferon or MEK-inhibitor treatment

**DOI:** 10.1186/s40425-019-0769-8

**Published:** 2019-11-18

**Authors:** Alice Newey, Beatrice Griffiths, Justine Michaux, Hui Song Pak, Brian J. Stevenson, Andrew Woolston, Maria Semiannikova, Georgia Spain, Louise J. Barber, Nik Matthews, Sheela Rao, David Watkins, Ian Chau, George Coukos, Julien Racle, David Gfeller, Naureen Starling, David Cunningham, Michal Bassani-Sternberg, Marco Gerlinger

**Affiliations:** 1 Translational Oncogenomics Lab, Centre for Evolution and Cancer, The Institute of Cancer Research, 237 Fulham Road, London, SW3 6JB UK; 2 Department of Oncology UNIL CHUV, Ludwig Institute for Cancer Research, University of Lausanne, 1005 Lausanne, Switzerland; 3 Vital IT, Swiss Institute of Bioinformatics, 1015 Lausanne, Switzerland; 4 Tumour Profiling Unit, The Institute of Cancer Research, 237 Fulham Road, London, SW3 6JB UK; 5 GI Cancer Unit, The Royal Marsden Hospital, Fulham Road, London, SW3 6JJ UK; 6 Swiss Institute of Bioinformatics (SIB), 1015 Lausanne, Switzerland

**Keywords:** Patient derived organoids, Colorectal cancer, Neoantigens, Immunogenicity, Human leukocyte antigen, Antigen presentation, Immunotherapy, Mass spectrometry

## Abstract

**Background:**

Patient derived organoids (PDOs) can be established from colorectal cancers (CRCs) as in vitro models to interrogate cancer biology and its clinical relevance. We applied mass spectrometry (MS) immunopeptidomics to investigate neoantigen presentation and whether this can be augmented through interferon gamma (IFNγ) or MEK-inhibitor treatment.

**Methods:**

Four microsatellite stable PDOs from chemotherapy refractory and one from a treatment naïve CRC were expanded to replicates with 100 million cells each, and HLA class I and class II peptide ligands were analyzed by MS.

**Results:**

We identified an average of 9936 unique peptides per PDO which compares favorably against published immunopeptidomics studies, suggesting high sensitivity. Loss of heterozygosity of the HLA locus was associated with low peptide diversity in one PDO. Peptides from genes without detectable expression by RNA-sequencing were rarely identified by MS. Only 3 out of 612 non-silent mutations encoded for neoantigens that were detected by MS. In contrast, computational HLA binding prediction estimated that 304 mutations could generate neoantigens. One hundred ninety-six of these were located in expressed genes, still exceeding the number of MS-detected neoantigens 65-fold. Treatment of four PDOs with IFNγ upregulated HLA class I expression and qualitatively changed the immunopeptidome, with increased presentation of IFNγ-inducible genes. HLA class II presented peptides increased dramatically with IFNγ treatment. MEK-inhibitor treatment showed no consistent effect on HLA class I or II expression or the peptidome. Importantly, no additional HLA class I or II presented neoantigens became detectable with any treatment.

**Conclusions:**

Only 3 out of 612 non-silent mutations encoded for neoantigens that were detectable by MS. Although MS has sensitivity limits and biases, and likely underestimated the true neoantigen burden, this established a lower bound of the percentage of non-silent mutations that encode for presented neoantigens, which may be as low as 0.5%. This could be a reason for the poor responses of non-hypermutated CRCs to immune checkpoint inhibitors. MEK-inhibitors recently failed to improve checkpoint-inhibitor efficacy in CRC and the observed lack of HLA upregulation or improved peptide presentation may explain this.

## Introduction

Immunotherapy with immune-checkpoint inhibitors (ICIs) is highly efficacious in microsatellite unstable (MSI) colorectal cancers (CRCs) but ineffective in microsatellite stable (MSS) CRCs [1, 2]. MSI tumors are deficient for DNA mismatch-repair mechanisms, resulting in high somatic mutation and neoantigen loads. Neoantigens are human leukocyte antigen (HLA)-binding peptides that encompass somatic mutations and they are considered a key substrate that enables T-cells to recognize tumor cells as foreign. In contrast to a mean of 1158 non-silent mutations in MSI CRCs, MSS tumors only harbor 123 mutations on average [3] which may explain poor ICI sensitivity. Yet, computational algorithms that consider the binding strength of mutated peptides to HLA Class I (HLA-I) molecules predicted that many MSS CRCs harbor over 100 mutated neoantigens [4]. This high number of predicted neoantigens contrasts with the poor senstivity of MSS CRCs to ICIs.

Mass spectrometry (MS) immunopeptidomics is an alternative method that directly assesses the repertoire of HLA-presented peptides and neoantigens. However, immunopeptidomics requires large quantities of material (usually > 1 g) [5–7], exceeding the amount that can be recovered from biopsies. Furthermore, the stromal content of CRCs can be high. As HLA-I molecules are expressed on cancer and stromal cells, the admixture of peptides from stromal cells makes it difficult to discern the cancer immunopeptidome.

Patient derived organoids (PDOs) can be established from CRC specimens, including even small biopsies, with success rates of up to 90% reported [8, 9]. Moreover, PDOs can be grown from patient tumors that match the stage and the pre-treatment histories of CRCs in which ICIs have been tested in clinical trials [1]. PDOs can be grown prospectively from patients undergoing treatment, permitting drug screening and correlative analyses.

Our aim was to develop culture techniques for CRC PDOs that enable analysis by MS to directly measure mutated neoantigens and to compare the results against computational predictions. PDOs are usually cultured in a 3D matrigel matrix which is expensive and laborious. We recently developed a method that grows PDOs attached to the surface of conventional plastic culture vessels in media supplemented with only 2% matrigel which overlays PDO cells and can be easily removed with the media [10]. Here, we show this enables large-scale expansion of PDOs to several hundred million cells, sufficient for in-depth immunopeptidomic analyses.

A further unique advantage of PDOs is the ability to investigate how perturbation influences the immunopeptidome. IFNγ is a key cytokine secreted by immune cells that can induce increased expression of HLA-I and II, and of immunoproteasome genes PSMB8, − 9, and − 10 in cancer cells [11], which may improve neoantigen processing and presentation. Genetic inactivation of IFNγ-signaling in cancer cells has been associated with failure of the immune system to clear cancer cells in murine models, and recently with ICI resistance [12, 13], supporting its clinical relevance.

Trametinib is an inhibitor of the mitogen-activated protein kinase (MAPK) pathway that inhibits MEK downstream of RAF kinases. This pathway is activated through genetic alterations, including mutations in *KRAS* or *BRAF* [14] in the majority of CRCs and MEK-inhibitor treatment has been shown to increase HLA expression [15]. Based on these results, MEK-inhibitors have been administered with ICI in a clinical trial in CRC but the combination was ineffective [16].

We first applied MS immunopeptidomics to five untreated PDOs, and subsequently investigated the effects of IFNγ and of the MEK-inhibitor trametinib on the neoantigen landscape. We further compared the results to computational predictions to investigate concordance.

## Methods

### Patients and samples

The establishment of the MSS CRC PDOs from the Prospect C, Prospect R (Chief investigator: D. Cunningham, UK national ethics committee approval numbers: 12/LO/0914 and 14/LO/1812, respectively) and the FOrMAT trials (Chief investigator: N. Starling, UK national ethics committee approval number 13/LO/1274) has previously been described [10]. All patients had provided written informed consent before trial inclusion.

### PDO culture and treatment

Establishing PDOs from tumor fragments required an average of 12 weeks and transition of PDOs from 3D to 2% matrigel culture, 5 weeks. For MS, PDOs were expanded over 8–16 weeks in DMEM/F12 media with 20% fetal bovine serum, Glutamax, 100 units/ml penicillin/streptomycin and 2% matrigel. Cells were changed into fresh media supplemented with DMSO, 30 nM/mL trametinib (Cayman Chemical) or 600 ng/mL IFNγ (R&D Systems) and left for 48 h. Cells were harvested with TrypLE express (ThermoFisher). PDOs were cultured identically for Western blots and flow cytometry.

### Exome sequencing

Sequencing libraries were prepared from > = 500 ng DNA from PDOs and matched blood using the Agilent SureSelectXT Human All Exon v5 kit according to the manufacturer’s protocol. Paired-end sequencing was performed on an Illumina HiSeq2500 with a target depth of 100x.

### Somatic mutation and copy number aberration analysis

Mutation and copy number calling have been described previously [11]. The cross-normal filter described in the ‘somatic mutation analysis’ methods section was replaced by simple cutoffs: Mutation calls with a minimum variant frequency of 10% and 6 variant reads in PDOs and a variant frequency ≤ 2.5%, a minimum depth ≥ 25 and ≤ 5 variant reads in the matched germline were retained. Indels were called with Platypus at depth > =15. Mutations with a cancer cell fraction [17] > 0.7 were considered clonal.

### HLA typing and mutation calling

4-digit HLA typing was performed with the TruSight HLA v2 Panel on a MiniSeq (Illumina). HLA allotypes were entered into the shell_call_hla_mutations_from_type script with POLYSOLVER [18].

### RNA-sequencing

3′-RNA-sequencing analysis of the five PDOs with the Lexogen Quantseq 3′ kit has been described previously [10] and we re-analyzed this dataset. We applied 3′-sequencing to RNA from PDOs treated with 600 ng/mL IFNγ or DMSO. The BlueBee cloud platform was used to normalize the data.

### Western blotting

Cell lysis was performed using NP-40 buffer with protease and phosphatase inhibitors (Sigma). Primary antibodies for p-ERK (Cell Signalling, #9101), ERK (Cell Signalling, #9102), and β-tubulin (Abcam #ab108342) were used. Detection was performed with an HRP-labelled secondary antibody (GE Healthcare) and ECL prime (GE Healthcare).

### HLA quantification by flow cytometry

HLA expression was assessed using the QIFIKIT quantitative flow cytometry assay (Agilent) according to the manufacturer’s instructions. Pan-HLA-A/B/C (BioLegend, W6/32), pan-HLA-DR/DP/DQ (BioLegend, Tü39), IgG2aκ isotype control (BioLegend, MOPC-173) were used.

### Purification of HLA peptides, LC-MS/MS analysis

Each PDO cell pellet (biological replicate, 3.85 × 10^7^–1X10^8^ cells/pellet) was split into two technical replicates that were processed as previously described [7]. See Supplementary Methods for details.

### Analysis of MS immunopeptidomics data

The ‘match between runs’ analysis was applied for all replicates and available treatment conditions, separately per PDO line, and separately between HLA-I and HLA-II samples. For the analysis of unique identified peptide sequences, we utilized a simple binary criterion of present or absent. A peptide was only defined as present if it was detected in both technical replicates of at least one biological replicate. All peptide lengths were considered when counting HLA-I-bound peptides, peptides > = 12aa when counting HLA-II-bound peptides. The raw MS intensity values were log2-transformed. As described [7], for differential expression analyses the Perseus platform [19] was used for ‘width normalization’, and missing values were imputed by random selection of values from a Gaussian distribution with a standard deviation of 20%. This provided intensity values in the range of − 10 to + 10, centered around 0. Differential expression was assessed from normalized data with a False Discovery Rate (FDR) *p*-value ≤0.05 and a fold change of ≥2 considered significant. In IFNγ-treated samples, genes from the HALLMARK_INTERFERON_GAMMA_RESPONSE gene set from GSEA [20] were highlighted and chymotrypsin-like ligands (defined as ending in “A”, “F”, “I”, “L”, “M”, “V”, “Y”) were assessed separately. HLA-II motif deconvolution is described in Supplementary Methods.

### Correlation of median peptide intensities between HLA-matched PDOs

The median non-normalized MS intensity values for peptides from two HLA-matched PDOs were plotted against each other, excluding peptides that were only present in one PDO.

### Correlation of gene expression and peptide presentation

The mean log2 gene expression of the 5 organoids was plotted against the mean normalized peptide appearance. Normalized peptide appearance was defined as the number of peptides from a gene that was detected by MS, divided by the protein length of that gene.

### Prediction of NetMHC percentile ranks from MS-detected peptides

All HLA-I MS-detected peptides were entered into NetMHCpan4.0 [21]. The HLA allotypes determined for each PDO line were selected for. Eluted ligand likelihood (ELL) predictions were used; the lowest ELL rank found for each peptide across all HLA allotypes was selected for further analysis.

### Computational prediction of neoantigens

Neoantigen sequences were predicted from somatic mutations (including non-silent substitutions and indels, but not splice-site mutations or stop-gains) as described [22] and ELL percentile rank scores were generated with NetMHCpan4.0 by running all neoantigen for each PDO against all corresponding HLA-I allotypes. For predicted strong binders, we selected core peptides with a percentile rank < 0.5%.

### Statistics

corr.test RStudio v3, was used to assess correlation, and paired t-tests with FDR multiple testing correction (GraphPad Prism) at 5% was used for differential expression analysis.

## Results

We previously described the propagation of PDOs from biopsies of one chemotherapy-naïve (CRC-08) and from four chemotherapy-resistant metastatic CRCs (CRC-01, − 03, − 04, − 05) [10]. Exome sequencing revealed 78–209 non-silent somatic mutations per PDO and driver mutations (Table 1), which are typical for MSS CRCs [3, 4]. 93% of all mutations were clonal. Several mutations on chromosomes that showed loss of heterozygosity (LOH) had variant allele frequencies between 99 to 100%, indicating that these were highly pure cancer cell populations without significant stromal cell components (mutation calls and variant allele frequencies: Additional file 1**:** Table S1, copy number profiles: Additional file 1**:** Figure S1). PDOs were expanded over 8–16 weeks using media supplemented with 2% matrigel to at least 200 million cells, followed by harvesting and snap freezing of at least two biological replicates with 100 million cells/replicate. The four fastest growing PDOs were expanded again and between 3 and 6 replicates were treated with 600 ng/ml IFNγ or 30 nM of trametinib for 48 h. The higher number replicates were expanded to compensate for potential cell death during treatment. However, this was modest, with median viability at the point of harvest ranging between 82 and 96% for the 4 treated PDOs, and all available cells were used for MS immunopeptidomics (Additional file 1**:** Table S2).

**Table 1:** Clinical characteristics of donor and mutation load in the 5 PDOs

	CRC-01	CRC-03	CRC-04	CRC-05	CRC-08
Age at biopsy (years)	60	61	59	52	51
Sex	male	female	female	male	male
Stage	IV	IV	IV	IV	IV
Prior chemotherapy	Yes	Yes	Yes	Yes	No
Non-silent mutation load	208	106	89	180	78
APC	p.Y935X, p.S1411 fs	p.R876X	p.S1356X	p.Q1367X	p.Y1075fs
TP53		p.G245S	p.T284 fs	p.R210X	p.R205C
KRAS	p.G12C	Amplification	p.A18D	p.G12D	p.G12D
TCF7L2		p.F105 fs			
SMAD4			p.G365R		

### Mass spectrometry identification of HLA-I ligands

**Fig. 1:**
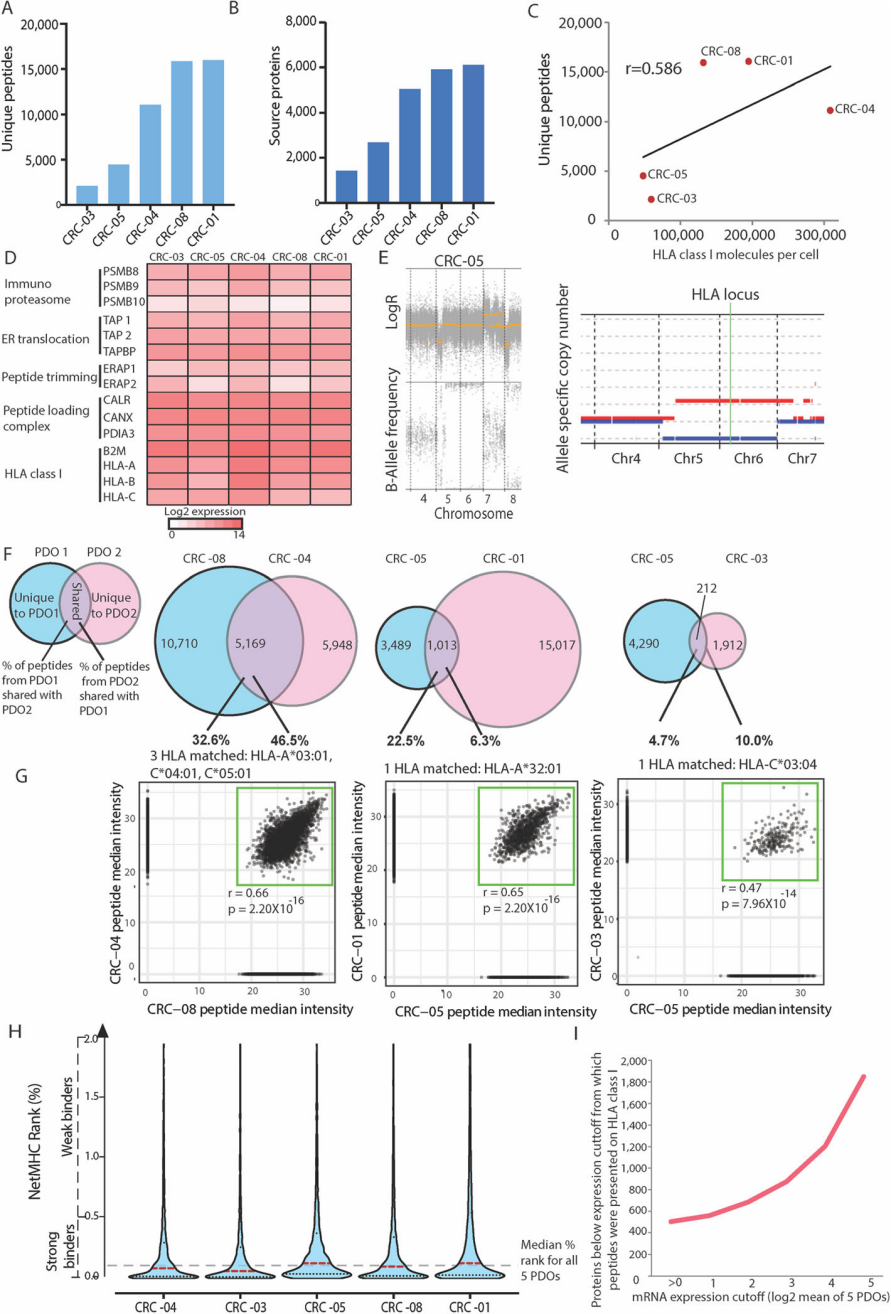
HLA-I immunopeptidome in five PDOs. **a** Number of unique peptides detected per PDO. **b** Number of source proteins to which peptides from A uniquely mapped. **c** Correlation of HLA-I molecules per cell (assessed by flow cytometry) against the number of unique peptides for all five PDOs. The Pearson correlation coefficient is shown. **d** RNA expression of genes involved in antigen processing and presentation on HLA-I. **e** DNA copy number data generated from exome sequencing of PDO CRC-05. **f** Venn diagrams showing the concordance and discordance of all peptides between pairs of PDOs which share the indicated HLA-I allele. Venn diagrams were re-scaled so the area represents the peptide numbers in each segment. **g** Comparison of the normalized peptide intensity of PDOs that share HLA-I alleles. **h** Violin plot of percentile ranks predicted by NetMHCpan4.0 for all MS identified peptides from panel A to the autologous HLA molecules per PDO. Dashed lines show the median for each PDO (red) and the overall median (black). **i** Number of MS detected peptides expressed at or below the indicated RNA expression value

We first analyzed how many peptides eluted from HLA-I molecules were detected by MS in each untreated PDO, by counting all unique peptide sequences that were identified in at least one biological replicate. Between 2124 and 16,030 HLA-I-bound peptides were identified across the 5 PDOs (Fig. 1a). The highest numbers were identified in CRC-01 (16,030 peptides) and CRC-08 (15,909 peptides). In CRC-01 and CRC-08, peptides originated from 6124 and 5928 source proteins, respectively (Fig. 1b). The mean number of unique HLA-I-presented peptides identified were 9936 per PDO. This exceeded the numbers seen in previous studies that applied similar MS-based immunopeptidomics techniques; for example, peptides in cell lines (mean: 7593/sample, range: 3293-13,696) [7], melanoma (mean: 3144/sample, range: 121–23,971) [5], ovarian (median: 1381/sample, range: 183–4289) [23] or CRC tumor samples (mean: 1171 peptides/cancer, range: 322–2407) [24]. Suggesting that our approach using PDOs for HLA-I-peptidome detection compares favorably in terms of sensitivity.

### Molecular factors influencing the immunopeptidome complexity

Our results showed a 7.5-fold variation in the number of peptides between these five PDOs and we sought to investigate the molecular determinants of this variability. We first measured the number of HLA-I molecules on the cell surface of each PDO by quantitative flow cytometric analysis. 48,202–308,847 (mean: 148,789) HLA-I molecules per cell were present in these PDOs (Fig. 1c). This showed a good correlation with the number of identified HLA-I peptides (Pearson correlation coefficient: 0.586, Fig. 1c), indicating that the number of cell surface HLA molecules influences the complexity of the immunopeptidome.

Genetic inactivation or impaired expression of HLA genes or genes encoding for the antigen processing and presenting machinery have been identified as a cause of reduced antigen presentation in multiple cancer types, including CRC [25, 26]. We hence assessed exome sequencing data for evidence of mutations or copy number aberrations in essential genes for antigen processing/HLA-I presentation [27] (listed in Fig. 1d). No mutations were found in these genes in any of the five PDOs. However, we identified LOH of chromosome 6, which harbors the HLA locus, in CRC-05 (Fig. 1e). LOH of all three HLA-I genes was independently confirmed by molecular HLA-typing (Additional file 1**:** Table S3). The restricted diversity of HLA alleles likely explains the limited diversity of the peptide repertoire in this PDO. All other PDOs were heterozygous for all three HLA-I loci. Genetic analysis did not define a reason for the low peptide diversity or HLA-I surface expression in CRC-03 and we hence investigated the expression of the essential genes for antigen processing/presentation in RNA-sequencing data (Fig. 1d). This showed no loss of expression that could explain the low peptide or HLA numbers in CRC-03. This highlights the need to further investigate the molecular mechanisms regulating antigen presentation in cancer.

### Impact of HLA allotypes on peptide presentation

We next assessed the overlap in peptide presentation between PDOs which shared HLA alleles. CRC-04 and CRC-08 had HLA-A*03:01, HLA-C*04:01 and HLA-C*05:01 in common. 23.7% of all detected peptides in these two PDOs were identical, and 46.5% of all peptides found in CRC-04, which displayed the lower total number of peptides, were shared by CRC-08 (Fig. 1f). Up to 22.5% of peptides in CRC-05 were also detected in CRC-01 which had one identical HLA-A allele, and up to 10.0% were shared by CRC-03 and CRC-05 with a single matching HLA-C allele. We next used NetMHCpan computational HLA binding predictions to assess the overlap of just the peptides predicted to bind to shared HLA-I allotypes. A mean of 42.07% (range: 1.18–70.19%) of these peptides were shared between PDO pairs, whereas only a mean of 2.73% (range: 0.10–7.09%) of peptides predicted to bind to the non-shared HLA-I allotypes were in common between the PDO pairs (Additional file 1**:** Figure S2). The MS intensities of shared peptides were highly similar (Pearson correlation coefficient: 0.4682–0.6632, Fig. 1g). This confirms that HLA allotypes are a major determinant of peptide presentation in cancer cells of the same type.

### Predicted HLA-I percentile ranks of MS-detected peptides

We applied NetMHCpan [21] to all MS-identified peptides to establish whether this algorithm could accurately predict them to be binders of the specific HLA-I alleles in these PDOs. 78.1% of the 49,682 detected peptides had a predicted rank < 0.5% which defines strong binders, and 93.0% of all peptides had a rank < 2% which includes weak and strong binders for at least one of the HLA alleles within the originating PDO (Fig. 1h). The median percentile rank of all peptides from all five PDOs was 0.1115% (range of medians for individual PDOs: 0.06650–0.1372%). This shows that the NetMHCpan algorithm accurately classifies the majority of detected peptides as binders and provides strong independent support for the origin of these MS identified peptides from the HLA-I binding groove.

### Predicting peptide presentation by mRNA expression analysis

Gene expression levels have been statistically associated with HLA-I peptide presentation levels in previous studies [28, 29]. Gene expression data from RNA-sequencing showed a weak correlation with peptide abundance, confirming a similar relationship for PDOs (Additional file 1**:** Figure S3). We next investigated if there is a minimum mRNA expression below which peptides from a protein cannot be detected. Out of the 13,761 genes that were expressed across the 5 PDOs, at least one peptide was detected by MS from 8464 (61.5%). However, peptides from 502 proteins were identified by MS but were not detectably expressed at the mRNA level. This may be explained by mRNA expression levels below the detection limit of our RNA-sequencing assay, or these could be wrongly identified peptide sequences, which are close to the allowed error rate of 1%. When a higher mean log2 expression value was used as a cut-off, the number of proteins that were expressed below this cut-off, but from which peptides were presented, increased rapidly (Fig. 1i). This suggests that a simple classification of genes into those that are detectably expressed at the mRNA level may be most useful to predict which proteins can be presented by HLA-I molecules.

### MS identification of HLA-II ligands

**Fig. 2:**
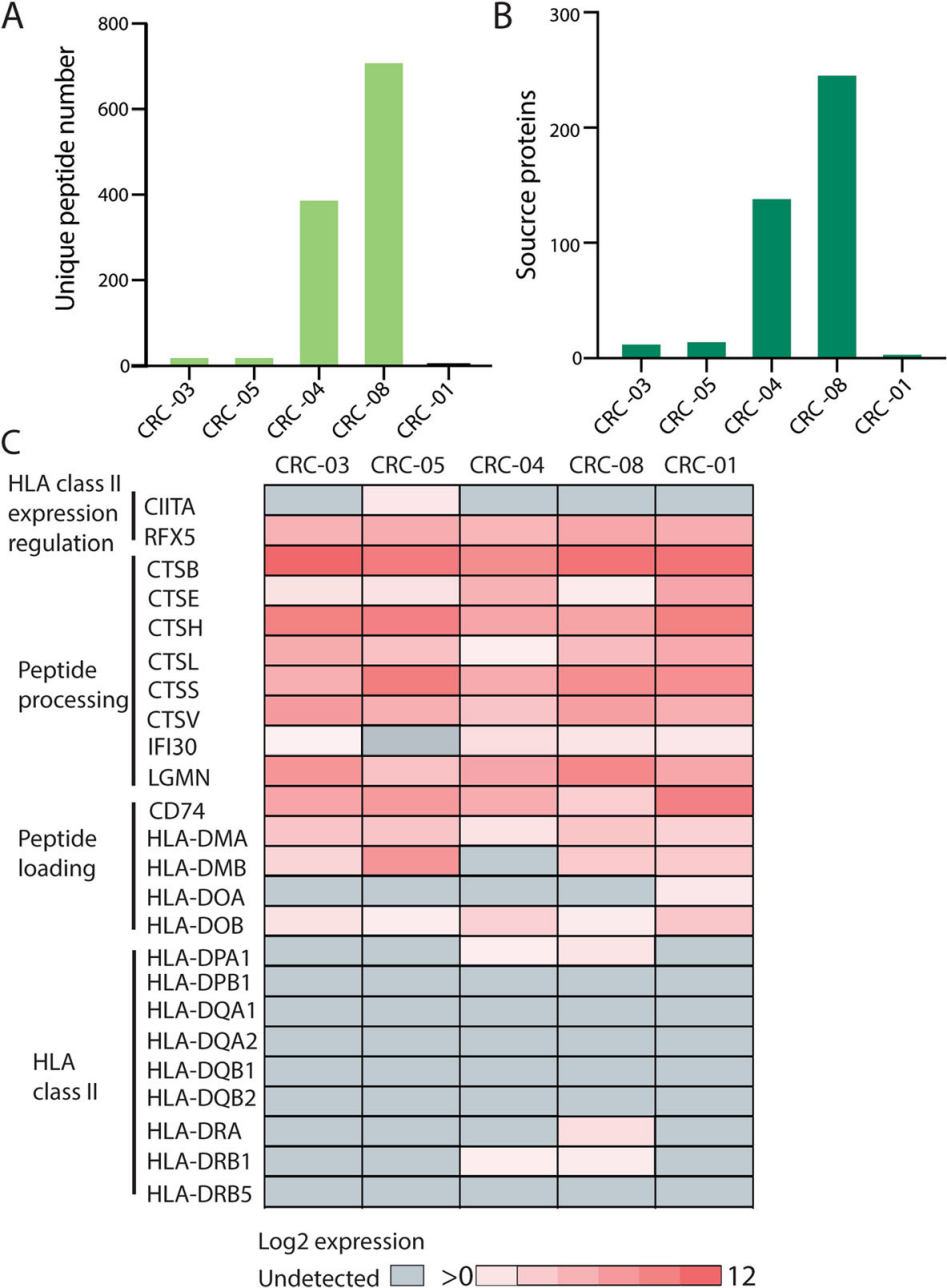
HLA-II immunopeptidome in five PDOs. **a** Number of unique peptides detected per PDO. **b** Number of source proteins to which peptides from A uniquely mapped. **c** RNA expression of genes involved in antigen processing and presentation on HLA-II

HLA-II molecules are mainly expressed on professional antigen presenting cells (APCs) and present peptides to CD4 T-cells [30], which have been shown to play a role in cancer cell recognition and killing [31]. Published data shows approximately 23% of CRCs express HLA-II, and this is associated with good prognosis [32]. 6–24 peptides were detected by MS on CRC-01, CRC—03, and CRC-05 (Fig. 2a-b). Three hundred ninety-two peptides from 140 source proteins, and 713 peptides from 247 source proteins were identified on CRC-04 and CRC-08, respectively (Fig. 2a-b). Cell surface HLA-II expression was below the limit of flow cytometric detection on all PDOs and RNA-sequencing showed no expression of HLA-II transcripts in CRC-01, CRC-03 and CRC-05 (Fig. 2c). Low-level HLA-II expression was detected by RNA-sequencing in both PDOs where we had identified HLA-II peptides (Fig. 2c), but neither expressed detectable transcripts of CIITA, the master regulator of HLA-II expression [33]. HLA-II expression despite undetectable CIITA levels may be explained by the limited sensitivity of RNA-sequencing, or perhaps by poor transcriptional control in CRC PDOs [34]. HLA-II peptide-binding motif deconvolution [35] revealed a clear motif for CRC-08 which fits to a known HLA-II motif (Additional file 1**:** Figure S4), supporting that these peptides were genuine HLA-II binders. Expression of HLA-II and peptide presentation were hence limited in our CRC PDOs, perhaps even lower than in CRC tumors due to the absence of any IFNγ-producing immune cells in PDO models.

### Neoantigen identification

**Fig. 3:**
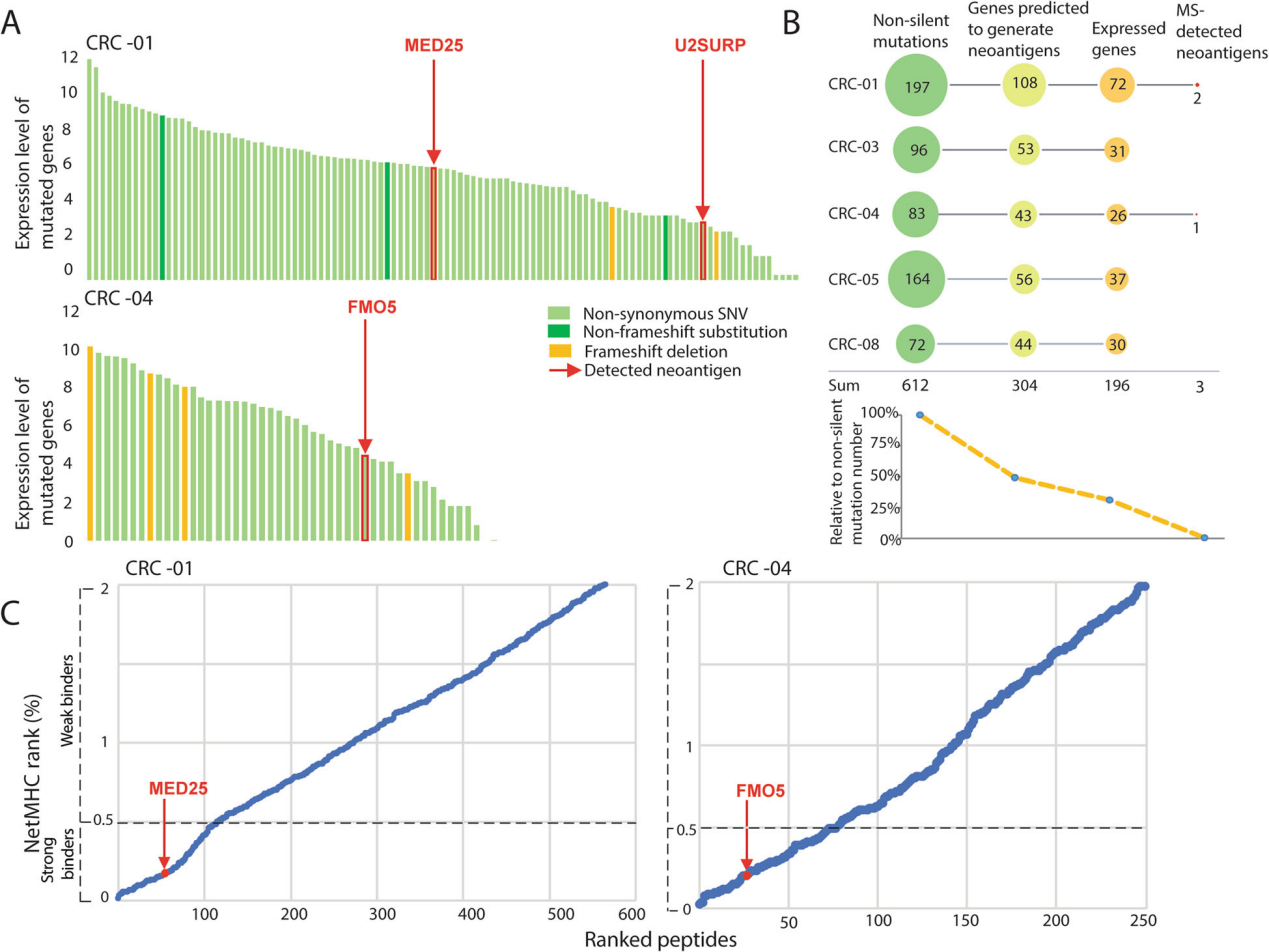
MS-detected and predicted neoantigens in five PDOs. **a** log2 gene expression of all genes harboring a mutation that encodes for an amino acid alteration. The three genes from which neoantigens were identified by MS are highlighted in red. **b** Number of mutations that encode for amino acid changes (missense, frame-shift and stop-loss mutations), genes predicted to generate strong binders predicted by NetMHCpan4.0 (defined as percentile rank below 0.5%), and strong binder-generating genes that are expressed, compared to MS-detected neoantigens. **c** HLA percentile rank from NetMHCpan4.0 for all predicted strong and weak HLA-binding neoantigen peptides in the two PDOs harboring MS-detected neoantigens. Predicted neoantigens were ordered from lowest to highest rank, with the predicted ranks of MS-detected neoantigens highlighted in red

The above results indicated that our immunopeptidomics approach performed robustly on PDOs and showed good sensitivity. We next questioned whether somatic mutation-encoded neoantigens could be detected by MS immunopeptidomics. Together, the five PDOs harbored 612 non-silent mutations that could generate predictable neoantigen sequences. All possible neoantigen sequences were used to assess MS spectra for evidence of neoantigen detection, applying a relaxed FDR of 5% as described [7]. This identified a total of only three neoantigens across the five PDOs (Table 2, MS spectra: Additional file 1**:** Figure S5), all encoded by clonal somatic mutations. CRC-01, the sample with the highest individual mutation load, harbored two mutations that encoded for HLA-I-presented neoantigens: one 8-mer originating from a mutation in the *MED25* gene, and one 11-mer from a mutation in *U2SURP*. A third neoantigen, a 10-mer, was detected in CRC-04, encoded by a mutation in *FMO5*. No HLA-II-presented neoantigens were identified. Plotting the mRNA expression values for all mutated genes in these two PDOs showed that the neoantigen source-genes were only moderately expressed in comparison to many other mutated genes (Fig. 3a). Together, only 3/612 (0.49%) of all mutations encoded for detectable neoantigens (Fig. 3b). All three were encoded by missense mutations whereas no neoantigens from any of the 33 frameshift mutations across these five PDOs were detected.

**Table 2:** MS-detected neoantigens

PDO	Source gene	Peptid length (amino acids)	Mutation	WT Peptide	Neoantigen	WT detected	Lowest NetMHC rank (%) WT	Lowest NetMHC rank (%) neoantigen
CRC-01	*U2SURP*	11	T224R	IQEERDERHKT	IQEERDERHKR	no	75.8495	5.7765
CRC-01	*MED25*	8	K422 T	SVDANTKL	SVDANTTL	no	0.5336	0.1586
CRC-04	*FMO5*	10	S423 N	RYVESQRHTI	RYVENQRHTI	no	0.31911	0.2692

To compare immunopeptidomics results to computational neoantigen prediction, we generated HLA-I binding predictions for somatic mutations that result in protein changes as described [22]. 304/612 mutations (49.67%) were predicted to encode for at least one strong binder (binding rank < 0.5%) of HLA-I (Fig. 3b). In CRC-05, which showed LOH of the HLA locus, only 34.14% of somatic mutations were predicted to generate a strong binder, compared to a mean of 55.74% in the other PDOs.

NetMHCpan only predicted two of the MS-identified neoantigens to be strong binders; the 8-mer from MED25 and 10-mer from FMO5, with ranks of 0.16 and 0.27%, respectively. Based on their rank, these peptides appeared in the top 1/3 of all predicted neoantigens (Fig. 3c). The ranks of the corresponding wild-type peptides were higher than those of the three detected neoantigens and neither of these has been detected by MS. Furthermore, the rank values shifted from weak binder to strong binder for MED25 (Table 2).

As an mRNA expression level of zero was a strong predictor that a specific protein is not presented on HLA-I, we next removed mutations in all genes with zero expression. This reduced the number of candidate mutations which are predicted to encode for neoantigens to 196/612 (32.03%) of all mutations (Fig. 3b). Thus, HLA-I ligands from 2/196 (1.02%) of the mutations computationally predicted as binders from expressed genes were actually detected, alongside 1 peptide not predicted to be a binder. Together, this shows that the number of potential neoantigens in colorectal cancers that can be identified on the cell surface is very low, even when high-sensitivity MS is used.

### Expression of cancer/testis antigens on HLA-I and II

We furthermore questioned whether peptides derived from tumor associated antigens, such as cancer/testis antigens, could be detected in any of the 5 PDOs. Due to central tolerance not being fully developed against these peptide:HLA complexes [36], T-cells may be able to recognize these peptides when aberrantly expressed on cancer cells, which could contribute to cancer antigenicity. Interrogating our immunopeptidomics dataset against 59 cancer/testis antigens [37], we found that only 2 PDOs presented peptides encoded by any of these genes. One peptide that originates from FAM46D was identified on CRC-01, and one from SPANXN3 was detected on CRC-08, both detected on HLA-I. No cancer/testis antigens were detected on HLA-II.

### Impact of IFNγ treatment on the immunopeptidome

**Fig. 4:**
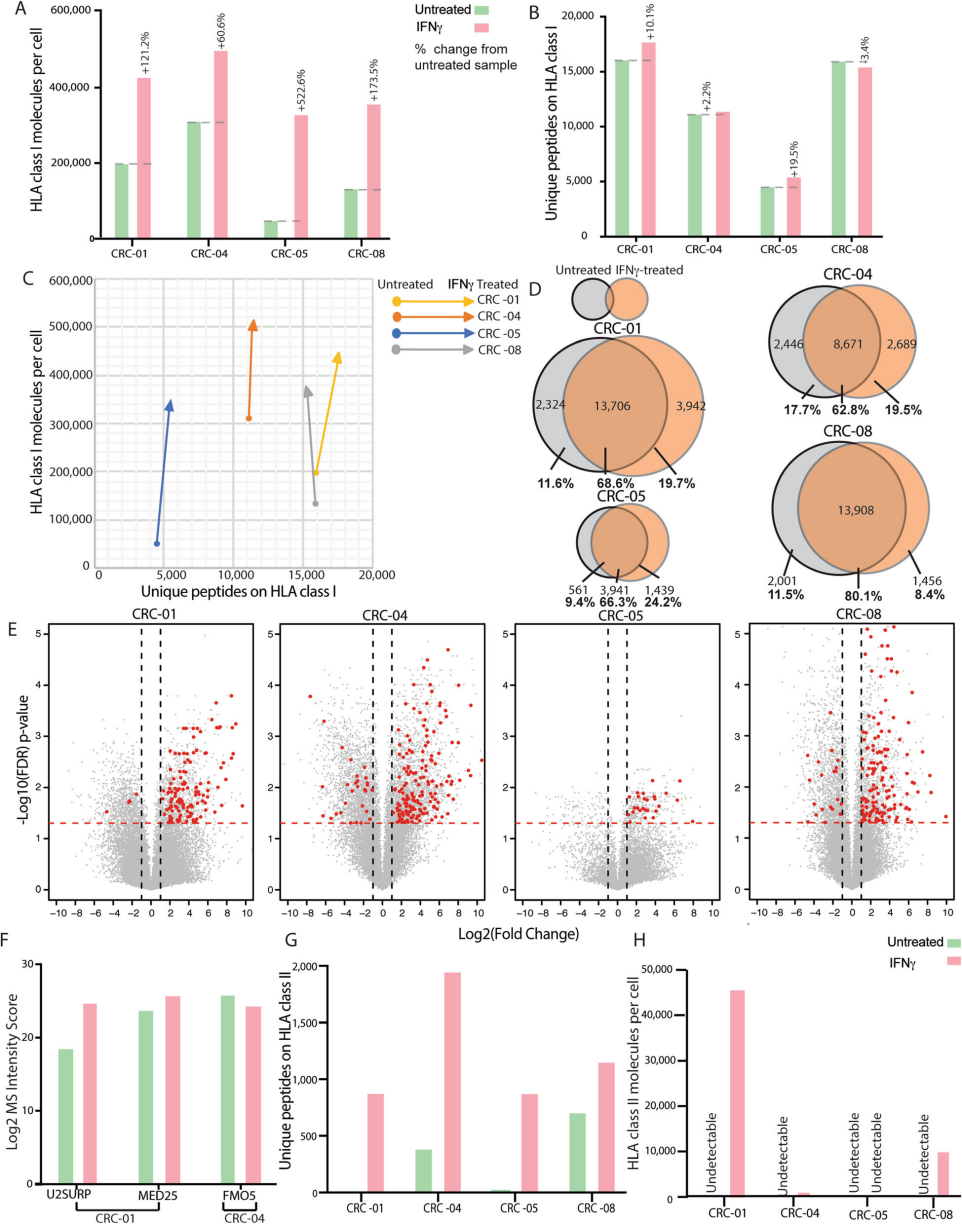
Changes of the immunopeptidome through IFNγ treatment (600 ng/ml for 48 h) in four PDOs. **a** Flow cytometric quantification of HLA-I molecules per cell with and without IFNγ treatment. **b** Number of unique peptides detected per PDO with and without IFNγ treatment. **c** Change in peptide diversity and HLA-I abundance with and without IFNγ treatment. **d** Venn diagram comparing the specific peptides detected in untreated and IFNγ-treated PDOs. Venn diagrams were re-scaled so the area represents the peptide numbers in each segment. **e** Volcano plots showing the fold change of normalized peptide abundance with IFNγ treatment. Known IFNγ-inducible genes which show a statistically significant (q < 0.05) fold change above +/− 2 are drawn in red. **f** MS intensities of neoantigens between untreated and IFNγ-treated conditions. **g** Number of unique peptides detected by MS on HLA-II molecules with and without IFNγ treatment. **h** Flow cytometric quantification of HLA-II molecules per cell with and without IFNγ treatment

Following treatment with IFNγ, HLA-I surface expression increased in all four treated PDOs (Fig. 4a), with a mean increase of 3.3-fold. Regardless of the number of HLA-I molecules in the untreated PDOs, HLA numbers rose to a similar level (330,108–495,981 molecules). Expression of IFNγ-regulated genes strongly increased following IFNγ treatment in all PDOs (Additional file 1**:** Figure S6A, Additional file 1: Table S4), confirming that IFNγ-signaling was preserved. Despite HLA-I upregulation and a 2.77–5.08-fold increase in mRNA expression of immunoproteasome genes (Additional file 1**:** Figure S6), we observed only modest changes in the numbers of peptides (Fig. 4b-c), with the largest increase in CRC-05 (+ 19.5%) and even a slight decrease in CRC-08 (− 3.4%).

However, differential presentation analysis revealed changes in the specific peptides that were presented. Only 69.45% of the peptides were shared between the untreated and IFNγ-treated samples on average (Fig. 4d). Comparison of peptide MS intensities furthermore showed up- or downregulation through IFNγ treatment; a mean number of 1371 peptides were upregulated at least 2-fold, and 1169 downregulated at least 2-fold (Fig. 4e). A mean of 119 peptides from IFNγ-inducible genes were significantly upregulated, compared with 13 that were downregulated. Moreover, the immunoproteasome has increased chymotrypsin-like activity compared to the constitutive proteasome [7] and we indeed observed an increased presentation of chymotrypsin-like ligands following IFNγ (Additional file 1**:** Figure S6B).

Importantly, we could not detect any additional neoantigens despite the described increase of antigen presentation efficiency through IFNγ [7]. All three neoantigens were identified again in IFNγ-treated PDOs and MS intensities of U2SURP- and of MED25-derived neoantigens increased with IFNγ treatment (Fig. 4f). An increased neoantigen abundance may be able to trigger a T-cell with a lower avidity TCR. The MS intensity of the FMO5 neoantigen decreased slightly.

IFNγ strongly increased the number of peptides presented on HLA-II, on all PDOs (Fig. 4g). Most of these peptides displayed known HLA-II binding motifs (Additional file 1**:** Figure S4), suggesting that the majority of them are bona fide HLA-II ligands. A corresponding increase of the number of HLA-II complexes (Fig. 4h), was demonstrated by flow cytometry in 3 PDOs, whereas HLA-II surface molecule numbers still remained below the detection limit for CRC-05 (Fig. 4h). These changes were accompanied by upregulation of CIITA and HLA-II genes (Additional file 1: Figure S7). No neoantigens were discovered on HLA-II following IFNγ treatment.

### Impact of trametinib treatment on the immunopeptidome

**Fig. 5:**
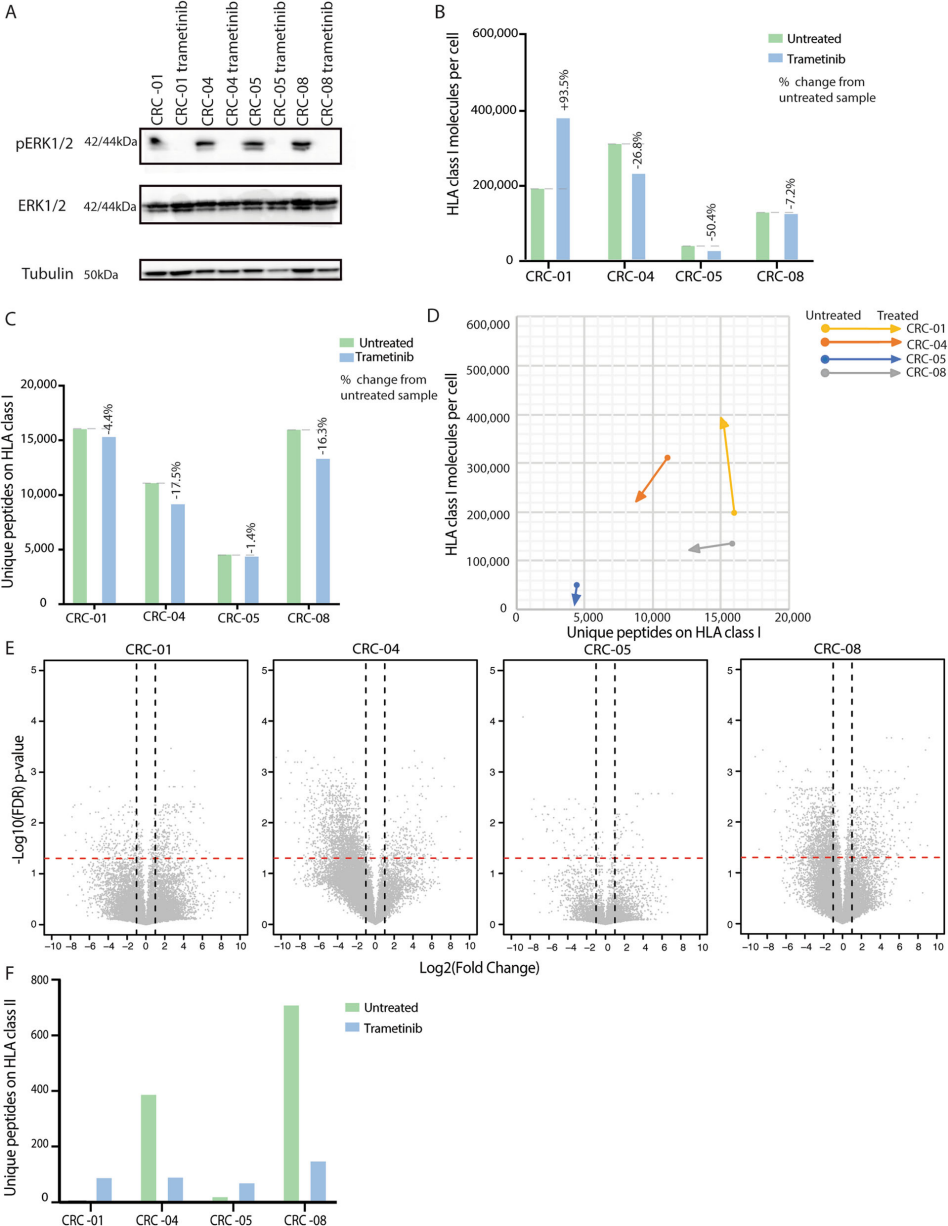
Changes of the immunopeptidome through trametinib treatment (30 nM for 48 h) in four PDOs. **a** Western blot showing inhibition of ERK phosphorylation (pERK) through trametinib. **b** Number of HLA-I molecules per cell with and without trametinib treatment. **c** Number of unique peptides presented on HLA-I with and without trametinib treatment. **d** Change in peptide diversity and HLA-I abundance with and without trametinib treatment. **e** Volcano plots showing the fold change of normalized peptide abundance with trametinib treatment. The dashed red lines indicate a q-value of 0.05 and vertical dashed lines fold changes exceeding +/− 2. **f** Number of unique peptides detected by MS on HLA-II molecules with and without trametinib treatment

48 h treatment with 30 nM of the MEK-inhibitor trametinib effectively blocked phosphorylation of the downstream effector ERK (Fig. 5a). This had no consistent effect on HLA-I surface expression, which increased in CRC-01 and slightly decreased in the other PDOs (Fig. 5b). Trametinib did not increase the number of HLA-I-presented peptides (Fig. 5c-d). CRC-04 showed the strongest fold-decrease in peptides at the cell surface with trametinib (Fig. 5e). Trametinib had variable effects on HLA-II-presented peptide numbers, which increased in two PDOs and decreased in the other two (Fig. 5f). No changes in HLA-II surface levels were detected. No additional neoantigens were detected in trametinib-treated PDOs.

## Discussion

Our study shows that MS-based immunopeptidomics is feasible from CRC PDOs. The ability to expand PDOs that were established from small biopsies to large cell numbers enabled the detection of a high number of distinct peptides, exceeding those found by other MS studies of large tissue samples and cell lines [5–7]. Together with the absence of stromal components, this suggests a comparatively high specificity for the analysis of the cancer cell immunopeptidome. MS immunopeptidomics has several limitations such as a finite detection sensitivity, biases towards the detection of peptides with high affinity to the HLA allotypes, peptides with good solubility in aqueous solution, and of peptides which can be well ionized [38]. We therefore cannot exclude the possibility that additional mutated neoantigens were presented but remained undetected with this experimental setup. However, individual HLA alleles have been estimated to be able to bind and present between 1000 and 10,000 peptides [28], suggesting that the 6 different HLA-I molecules in an individual can on average present ~ 30,000 distinct peptides. We identified up to 16,030 peptides per PDO and up to 3942 additional peptides were detected after IFNγ stimulation. This suggests that we sampled over 50% of the estimated peptide presentation capability in some of our PDOs.

Despite this, we only identified three mutated neoantigens in five PDOs that together harbored 612 non-silent somatic mutations. Neither IFNγ, nor MEK-inhibitor, promoted the presentation of additional MS-detectable neoantigens. Importantly, 4 of the PDOs were derived from metastatic tumors that were resistant to prior palliative chemotherapy. Their biology and mutational loads should hence represent some of the features of advanced and treatment refractory CRCs in which novel immunotherapy trials are usually undertaken. The sparse neoantigen landscape observed in all five MSS CRCs hence provides a potential explanation for the low efficacy of ICI in MSS CRCs [1]. A limitation of our work is the lack of an MSI PDO line as a positive control, and for comparison. Analysis of this immunotherapy-sensitive CRC subtype could be used to further validate PDO immunopeptidomics and provide insights into the quantity and quality of neoantigens that enable effective cancer immune recognition. Comparison to PDOs from pre-invasive MSS CRCs is desirable to assess whether they harbor higher neoantigen numbers than our PDOs from more advanced CRCs, which would indicate immunoediting as a mechanism of neoantigen loss [3, 39].

Our data contrasts with published data showing that neoantigen-specific T-cells were present among tumor infiltrating lymphocytes in 5 of 5 CRCs [40]. However, this study only assessed the specificity of T-cells against minigene-derived neoantigens presented on APCs, and did not assess whether the T-cells were also able to recognize autologous cancer cells. Autologous T-cells were not available for our PDOs, precluding in vitro T-cell recognition assays to assess whether the MS-identified neoantigens can be recognized by CD8 T-cells or whether T-cells can recognize PDOs without MS-detectable neoantigens. Such studies that combine PDO immunopeptidomics and functional T-cell assays will be the critical next step to further delineate the CRC neoantigen landscape.

Investigating non-mutated cancer/testis antigens only identified one peptide from each of two cancer/testis antigens (FAM46D, SPANXN3). However, only antibody responses have been described against these so whether they can elicit T-cell responses is unclear [41, 42].

The low number of neoantigens encoded by somatic mutations and of peptides from cancer/testis antigens are sobering as they indicate that endogenous immunogenicity may be low in metastatic and drug resistant CRCs. A similar scarcity of neoantigens in tumors with moderate mutation loads has recently been suggested by MS of hepatocellular carcinomas [43]. Both studies reveal that the HLA-I immunopeptidome only presents a small fraction of the protein-coding genome to CD8 T-cells. This highlights a need to assess neoantigens from other sources (e.g. T-cell epitopes associated with defects in antigen processing [44], fusion genes, de-repressed endogenous retroviruses, transposable elements, post-translationally-modified peptides, and from novel open reading frames [45]) or to develop novel immunotherapies that facilitate immune recognition despite a limited number of antigens. Bispecific antibodies or CAR-T-cells that target cell surface molecules which are overexpressed on cancer cells, such as CEA, are examples of such therapies.

Comparing MS immunopeptidomics data with neoantigen predictions using the NetMHCpan algorithm, which is one of the current gold standards, suggested over-prediction of neoantigens by computational analysis. 304/612 mutations (49.67%) were predicted to generate peptides that strongly bind autologous HLA-I and 196 of these were located in genes with detectable RNA expression. This contrasts with only 3 MS-detected neoantigens, constituting only 0.49% of all non-silent mutations. This highlights the need to improve the understanding of peptide processing and presentation.

A unique advantage of PDOs immunopeptidomics is the ability to analyze how drug treatment or cytokines influence the peptidome. IFNγ increased the number of HLA-I molecules at the cell surface in all four PDOs and of unique peptides in 2/4 PDOs. Together, the modest change in the number of distinct peptides despite the strong increase in HLA-I expression on the cell surface indicates that the diversity of the peptide repertoire remains restricted. This is likely due to the constraints of antigen processing and HLA allotype binding. Furthermore, the number of unique HLA-II-presented peptides strongly increased.

PDO immunopeptidomics could hence support the development of novel strategies to increase peptide and neoantigen presentation, alongside generating more MS training data, to improve epitope prediction algorithms [28, 35, 46]. This is particularly important for the ongoing development of mutanome-specific vaccines targeting predicted neoantigens [47, 48] as false positive predictions may lead to targeting of irrelevant epitopes.

MEK-inhibitor treatment did not consistently increase HLA expression or peptide presentation. This may explain the lack of efficacy of MEK-inhibitors in combination with a PD-L1 ICI in a recent clinical trial [16] which was partly based on the observation that MEK inhibition could increase HLA-I expression in a CRC mouse model [49]. Testing such strategies in PDOs, which may more accurately represent patient tumors than established cell lines or mouse models [10], may enable the pre-clinical validation of novel immunotherapy combinations before embarking on clinical trials.

## Conclusions

This study shows that MS immunopeptidomics of CRC PDOs is feasible and that it enables assessment of how in vitro perturbation alters antigen presentation. MS immunopeptidomics only identified a small number of neoantigens in PDOs. This may explain the poor activity of ICIs in MSS CRCs. Detailed insights into the CRC neoantigen landscape through PDO immunopeptidomics may be useful to improve neoantigen prediction technologies, personalized vaccine design, and to identify novel approaches to increase neoantigen presentation.

## Supplementary information

**Additional file 1.** Supplementary information

## Data Availability

RNA sequencing data of PDOs has been published in the supplementary material to [10]. Exome sequencing data has been deposited in the EGA archive with the submission ID EGAS00001003886. Access will be granted after signing an MTA which restricts dissemination of the data and any attempts to re-identify the patient donors. The mass spectrometry immunopeptidomics data have been deposited to the ProteomeXchange Consortium via the PRIDE [50] partner repository with the dataset identifier PXD014017.

## Abbreviations

APC: Antigen Presenting Cell
CRC: Colorectal Cancer
CTLA4: Cytotoxic T-lymphocyte–associated Antigen 4
ELL: Eluted Ligand Likelihood
FDR: False Discovery Rate
GSEA: Geneset Enrichment Analysis
HDAC: Histone Deacetylases
HLA: Human Leukocyte Antigen
ICI: Immune Checkpoint Inhibitor
IFNγ: Interferon Gamma
LC-MS/MS: Liquid Chromatography tandem Mass Spectrometry
MS: Mass Spectrometry
PDL1: Programmed Death-Ligand 1
PDO: Patient Derived Organoid
