# Supplementary material for: Immunopeptidomics of colorectal cancer organoids reveals a sparse HLA class I neoantigen landscape and no increase in neoantigens with interferon or MEK-inhibitor treatment
Source: J Immunother Cancer. 2019 Nov 18;7:309. doi: 10.1186/s40425-019-0769-8 (PMC6859637; doi:10.1186/s40425-019-0769-8)
Supplement: Supplementary file 1 — Additional file 1. Supplementary information [file 40425_2019_769_MOESM1_ESM.zip › Supplementary Information.pdf]

Supplementary material for Newey et al.

Table of contents:

Supplementary Methods *Page 2*

Purification of HLA-I and HLA-II peptides and LC-MS/MS analyses *Page 2*

Peptide identification from MS data *Page 4*

HLA-II motif deconvolution *Page 4*

Supplementary Table 2: Total cell numbers used and number of repeats *Page 5*

Supplementary Table 3: HLA typing of PDOs *Page 6*

Supplementary Figure 1: Copy number profiles generated from exome sequencing data of PDOs. *Page 7-8*

Supplementary Figure 2: Venn diagrams of peptide overlaps between shared and non-shared HLA allotypes between PDO pairs *Page 9*

Supplementary Figure 3: Correlation between gene expression and normalized protein appearance on HLA-I *Page 10*

Supplementary Figure 4: Motif deconvolution from HLA-II peptidomics data *Page 11*

Supplementary Figure 5: MS spectra of detected neoantigens *Page 12*

Supplementary Figure 6: Heatmaps and volcano plots of changes induced by IFNγ treatment *Page 13*

Supplementary Figure 7: Heatmap of changes in key genes for HLA-II peptide presentation induced by IFNγ treatment *Page 14*

References *Page 15*

Supplementary methods:

Purification of HLA-I and HLA-II peptides and LC-MS/MS analyses

Anti-pan-HLA-I and anti-HLA-II monoclonal antibodies were purified from the supernatant of HB95 (ATCC® HB-95™) and HB145 cells (ATCC® HB-145™), respectively, using Protein A-Sepharose 4B beads (Invitrogen). Antibodies were cross-linked to beads at a concentration of 5 mg of antibodies per 1 mL volume of beads with Dimethyl pimelimidate dihydrochloride (Sigma-Aldrich) in 0.2 M Sodium Borate buffer pH 9 (Sigma-Aldrich) at a final concentration of 20 mM for 30 minutes. The reaction was quenched by incubation with 0.2 M ethanolamine pH 8 (Sigma-Aldrich) for 2 hours. Cross-linked antibodies were kept at 4°C until use.

Shortly, lysis of cells was done at 4°C for 1 hour in PBS containing 0.25% sodium deoxycholate, 0.2 mM iodoacetamide, 1 mM EDTA, 1:200 Protease Inhibitors Cocktail (Sigma-Aldrich), 1 mM Phenylmethylsulfonylfluoride, 1% octyl-beta-D glucopyranoside. Cell lysates were cleared by centrifugation with a table-top centrifuge at 4°C at 14,200 rpm for 50 min. For the sequential purification of HLA-I and HLA-II, two stacked 96-well single-use micro-plates (3µm glass fiber, 10µm polypropylene membranes; Seahorse Bioscience) were used. The first contained cross-linked beads with anti HLA-I antibodies and the second contained the anti HLA-II cross-linked beads. The lysates were loaded on plates by gravity. Then the Waters Positive Pressure-96 Processor was employed and the plates were washed separately with 4 times 2 mL of 150 mM sodium chloride (NaCl) in 20 mM Tris-HCl pH 8, 4 times 2 mL of 400 mM NaCl in 20 mM Tris-HCl pH 8 and again with 4 times 2 mL of 150 mM NaCl in 20 mM Tris-HCl pH 8. Finally, the plates were washed twice with 2 mL of 20 mM Tris-HCl pH 8. Each plate was stacked on top of a Sep-Pak tC_{18} 100 mg Sorbent 96-well plate (Waters) equilibrated

with 1 mL of 80% acetonitrile (ACN) in 0.1 % TFA and with 2 mL of 0.1% TFA. The HLA and peptides were eluted with 500 µL 1% TFA into the Sep-Pak plate and then the Sep-Pak plates were washed with 2 mL of 0.1 % TFA. HLA-I and HLA-II peptides were eluted with 500 µL of 32% ACN in 0.1% TFA into a collection plate. Recovered peptides were dried using vacuum centrifugation and stored at -20°C.

Prior to MS analysis, HLA-I and HLA-II peptide samples were re-suspended in 9 µL of 0.1 % FA and 2/3 of the sample volume were placed in the Ultra HPLC autosampler. Each sample was measured in technical duplicates. HLA peptides were separated by an Easy-nLC 1200 (Thermo Fisher Scientific) coupled on-line to a Q Exactive HFX mass spectrometers (Thermo Fisher Scientific) with a nanoelectrospray ion source (Proxeon Biosystems). Self-packed 50 cm long (75 µm inner diameter) column with ReproSil-Pur C18-AQ 1.9 µm resin (Dr. Maisch GmbH) in buffer A (0.5% acetic acid). HLA-I and HLA-II peptides were eluted with a linear gradient of 2–30% buffer B (80% ACN and 0.5% acetic acid) at a flow rate of 250 nl/min over 125 min and 90 min, respectively.

MS spectra were acquired from m/z = 300-1650 in the Orbitrap with a resolution of 60’000 (m/z = 200) and ion accumulation time of 80 ms. The auto gain control (AGC) was set to 3e6 ions. MS/MS spectra were acquired on 10 most abundant precursor ions (if present) with a resolution of 15,000 (m/z = 200), ion accumulation time of 120 ms and an isolation window of 1.2 m/z. The AGC was set to 2e5 ions, dynamic exclusion to 20 s and a normalized collision energy (NCE) of 27 was used for fragmentation. The peptide match option was disabled. No fragmentation was performed for HLA-I peptides in case of assigned precursor ion charge states of four and above, and for HLA-II peptides, in case of assigned precursor ion charge states of one, and also from six and above.

Peptide identification

We searched the immunopeptidomics peak lists data with a PSM false discovery date of 1% with the MaxQuant platform (1) version 1.5.5.1 against a fasta file containing the human proteome (Homo_sapiens_UP000005640_9606, the reviewed part of UniProt, with no isoforms, including 21,026 entries downloaded in March 2017) and a list of 247 frequently observed contaminants. In addition, MaxQuant version 1.5.9.4 was used for a search against a customized reference database were somatic mutations were included, with a PSM false discovery rate of 5%. Peptides with a length between 8 and 25 AA were allowed. The second peptide identification option in Andromeda was enabled. The enzyme specificity was set as unspecific and no protein FDR was set. The initial allowed mass deviation of the precursor ion was set to 6 ppm and the maximum fragment mass deviation was set to 20 ppm. Methionine oxidation and N-terminal acetylation were set as variable modifications.

HLA-II motif deconvolution

HLA-II peptidomics samples that contained at least 500 peptides with a length of 12 amino acids and longer were submitted to motif deconvolution with MoDec(2). MoDec seeks to find common motifs shared by subsets of the peptides from a given sample. These motifs were shown to accurately represent the binding specificity of the HLA-II alleles in the sample(2). Peptides that do not fit a motif inferred by MoDec are assigned to a flat motif, and represent potential contaminants or peptides displayed on HLA-II molecules with few ligands in the sample. MoDec version 1.1 was run with the recommended parameters for HLA-II peptidomics (a minimum peptide length of 12 and multiple types of initial conditions with help of “--specInits" parameter). The optimal number of motifs was manually determined for each sample.

Supplementary table 2: Total cell numbers used and number of repeats

		CRC-01	CRC-03	CRC-04	CRC-05	CRC-08
**Untreated**	Biological replicates	2	3	2	2	3
	Total cell number	2x10^8	3x10^8	2x10^8	2x10^8	2x10^8
	Median viability	85%	74%	88%	92%	96%
**IFNγ**	Biological replicates	5	-	4	3	6
	Total cell number	3.205x10^8		4x10^8	3x10^8	6x10^8
	Median viability	82%		84%	87%	92%
**Trametinib**			-			
	Biological replicates	4		4	4	4
	Total cell number	4x10^8		4x10^8	4x10^8	4x10^8
	Median viability	96%		82%	88%	86%

Supplementary table 3: HLA typing of PDOs

	CRC-01	CRC-03	CRC-04	CRC-05	CRC-08
**HLA-A**	31:01	02:01	03:01	32:01	03:01
	32:01	29:02	24:02		29:01
**HLA-B**	14:01	15:01	18:01	40:01	35:03
	27:05	44:03	35:08		44:02
**HLA-C**	02:02	03:04	04:01	03:04	04:01
	08:02	16:01	05:01		05:01

Supplementary Figure 1: Copy number profiles generated from exome sequencing data of PDOs

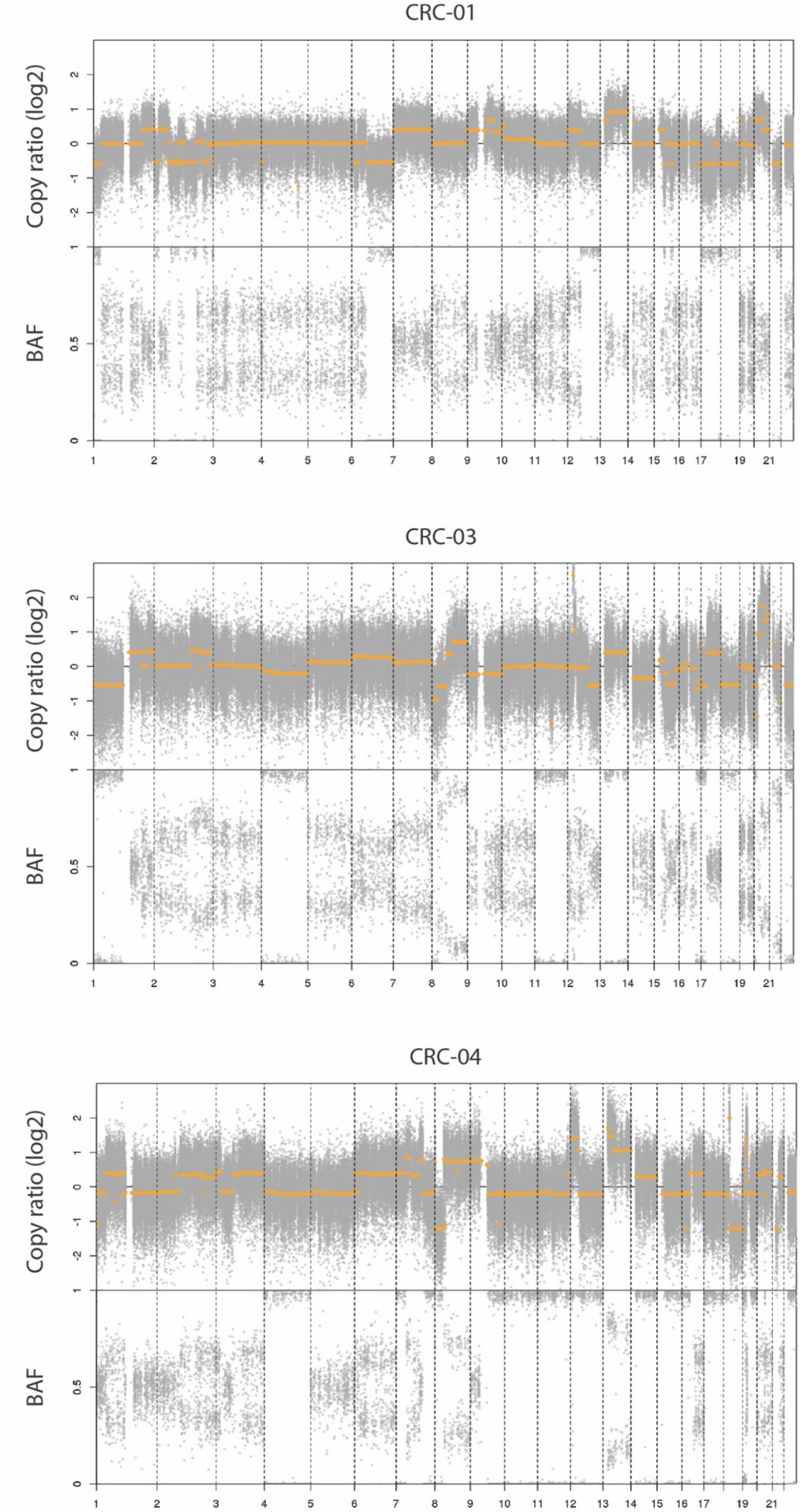

.

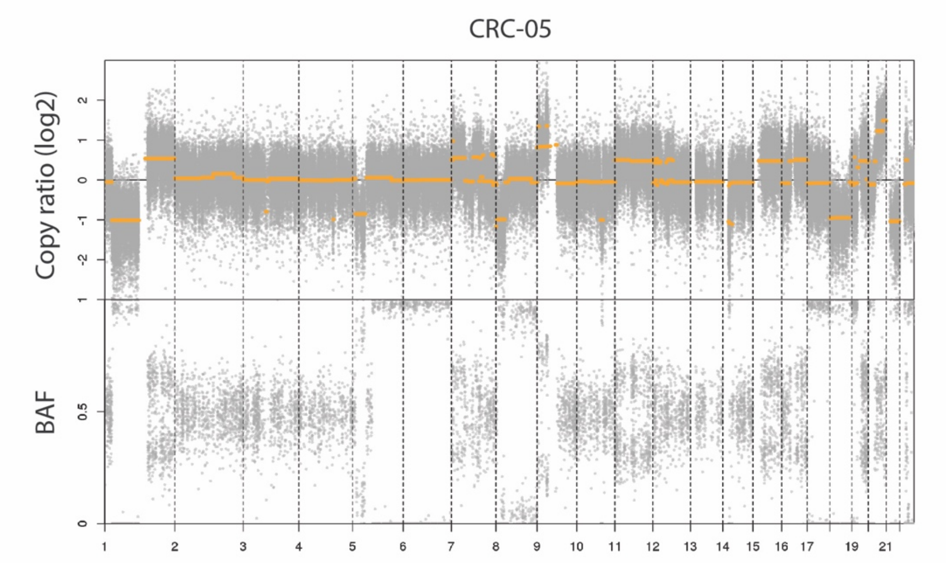
CRC-05
Copy ratio (log2)
BAF
1
2
3
4
5
6
7
8
9
10
11
12
13
14
15
16
17
19
21

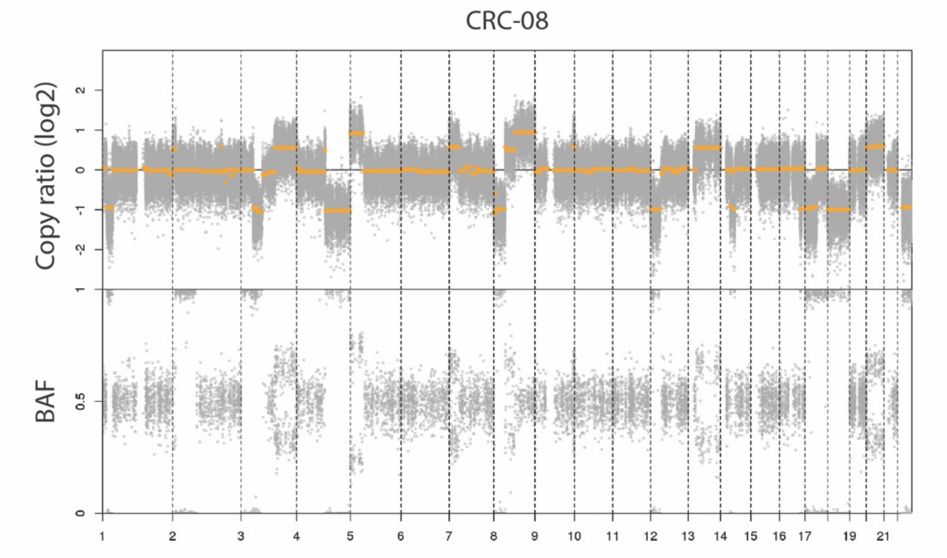
CRC-08
Copy ratio (log2)
BAF
1
2
3
4
5
6
7
8
9
10
11
12
13
14
15
16
17
19
21

Supplementary Figure 2: Venn diagrams of peptide overlaps between shared and non-shared HLA allotypes between PDO pairs. Venn diagrams showing the concordance and discordance of peptides between pairs of PDOs which share the indicated HLA-I alleles split into peptides predicted by NetMHCpan4.0 to bind to the matched HLAs (a threshold of a binding rank <2%) and peptides that are predicted to bind to unmatched HLAs; only peptides which were predicted to uniquely bind one HLA allotype were included.

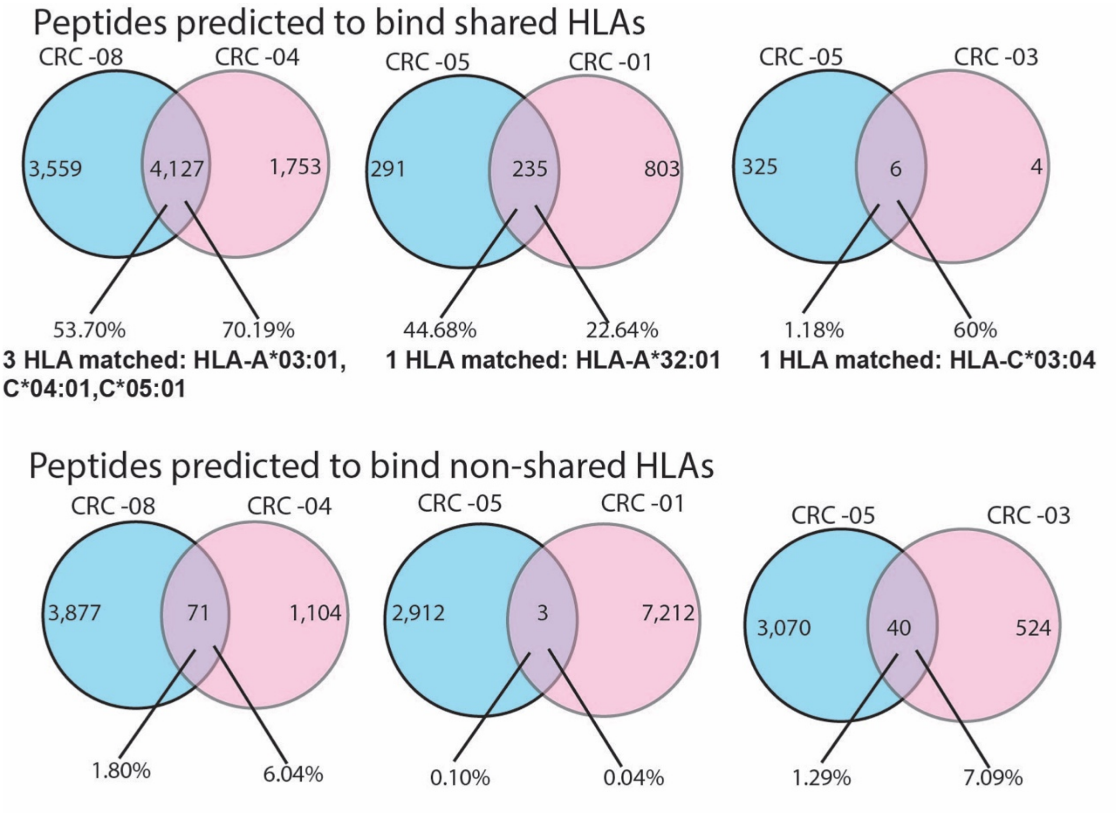

Supplementary Figure 3: Correlation between gene expression and normalized protein appearance on HLA-I

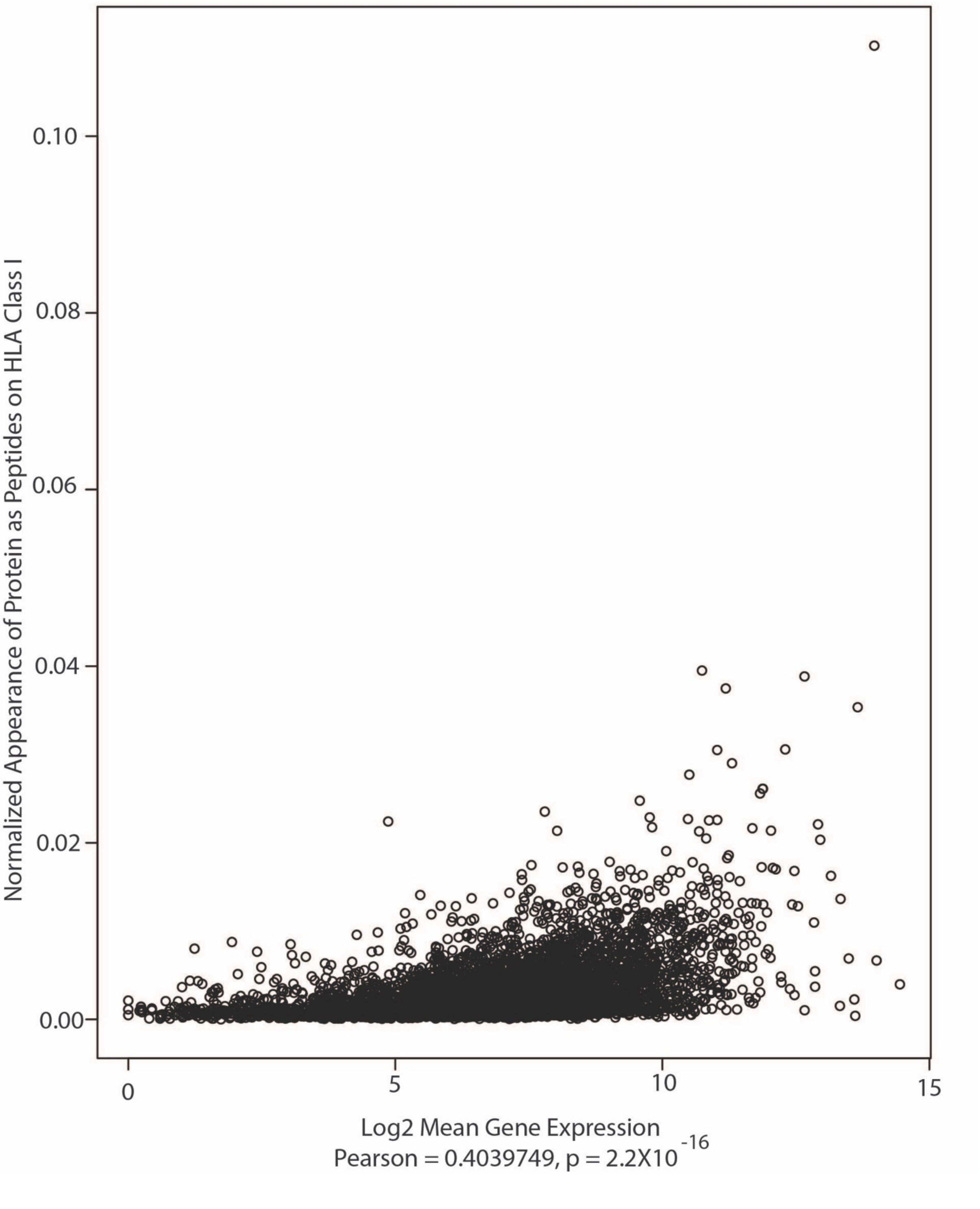

Supplementary Figure 4: Motif deconvolution from HLA-II peptidomics data. A: Motifs found after applying MoDec(2) for the HLA-II peptidomics from the PDOs. Percentages above each motif indicate the fraction of peptides from the given sample that were assigned to the motif; peptides that do not fit a motif inferred by MoDec are assigned to a flat motif, and represent potential contaminants or peptides displayed on HLA-II molecules with few ligands in the sample f. **B:** Known motifs from common HLA-II alleles, adapted from(2). Colored boxes show highly similar motifs between the samples A and corresponding alleles B; motifs without a coloured box could not be confidently attributed to the known motifs in B.

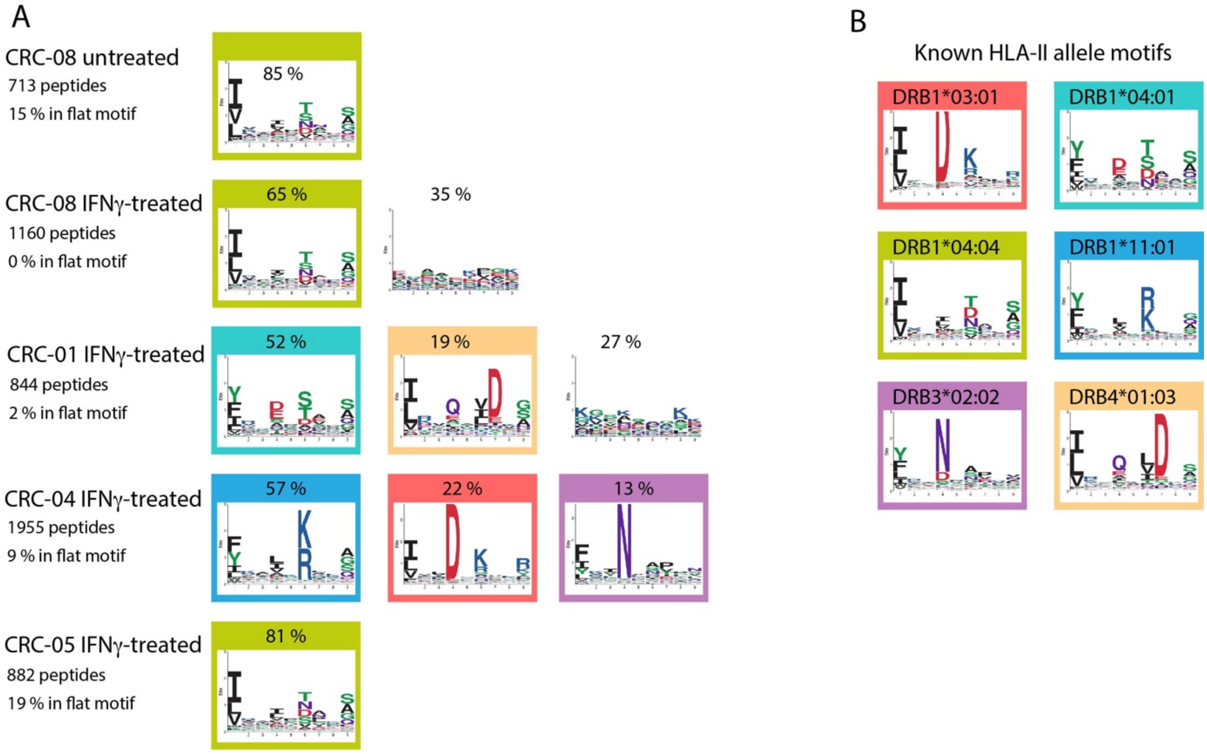

Supplementary Figure 5: MS spectra of detected neoantigens. Annotated MSMS fragmentation spectra of the three identified neoantigens including their MaxQuant identification score supporting the peptide spectrum match and the score difference to second best fit.

IQEERDERHKR ; U2SURP ; U2 snRNP-associated SURP motif-containing protein
MaxQuant identification score: 82 ; Delta score: 37

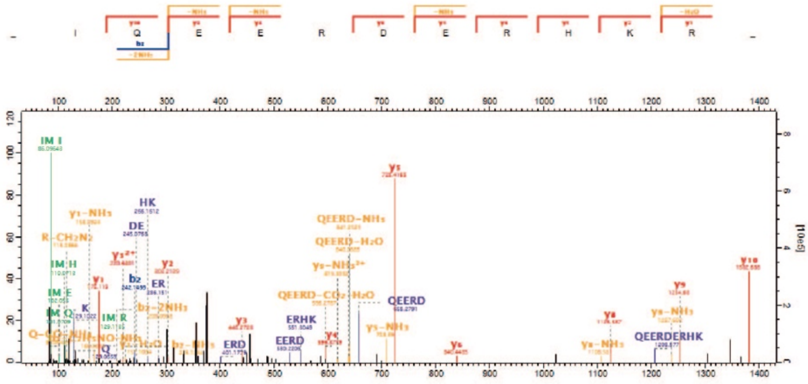

SVDANTTL ; MED25 ; Mediator of RNA polymerase II transcription subunit 25
MaxQuant identification score: 96 ; Delta score: 13

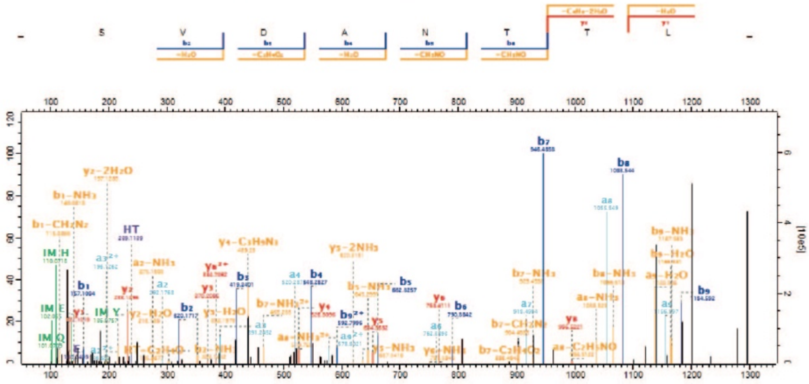

RYVENQRHTI ; FMO5 ; Dimethylaniline monooxygenase [N-oxide-forming] 5
MaxQuant identification score: 152 ; Delta score: 98

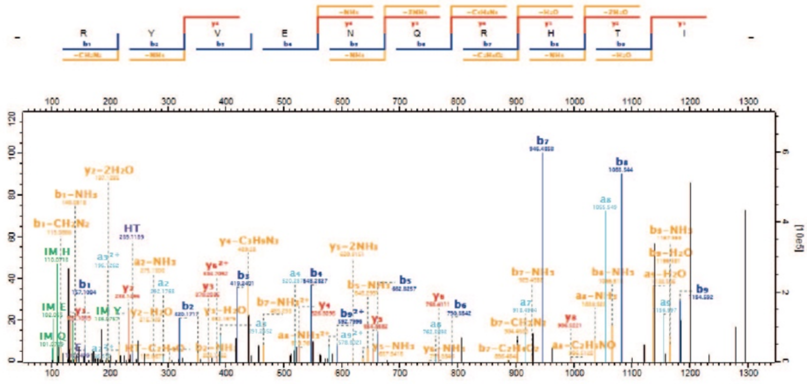

Supplementary Figure 6: Heatmap and volcano plots of changes induced by IFNγ treatment. **A:** Heatmap of key genes involved in peptide processing and HLA-I presentation, including immunoproteasome subunits PSMB8, PSMB9, PSMB10, to illustrate changes in expression between the untreated and IFNγ-treated conditions. Significant difference analysis performed with a paired t-test, followed by FDR analysis set to 5%. **B:** Volcano plots showing the fold change of normalized peptide abundance with IFNγ treatment with chymotrypsin-like ligands (defined as ending in "A", "F", "I", "L", "M", "V", "Y"). Numbers of peptides which show a statistically significant (q<0.05) fold change above +/-2 are highlighted above the volcano plots.

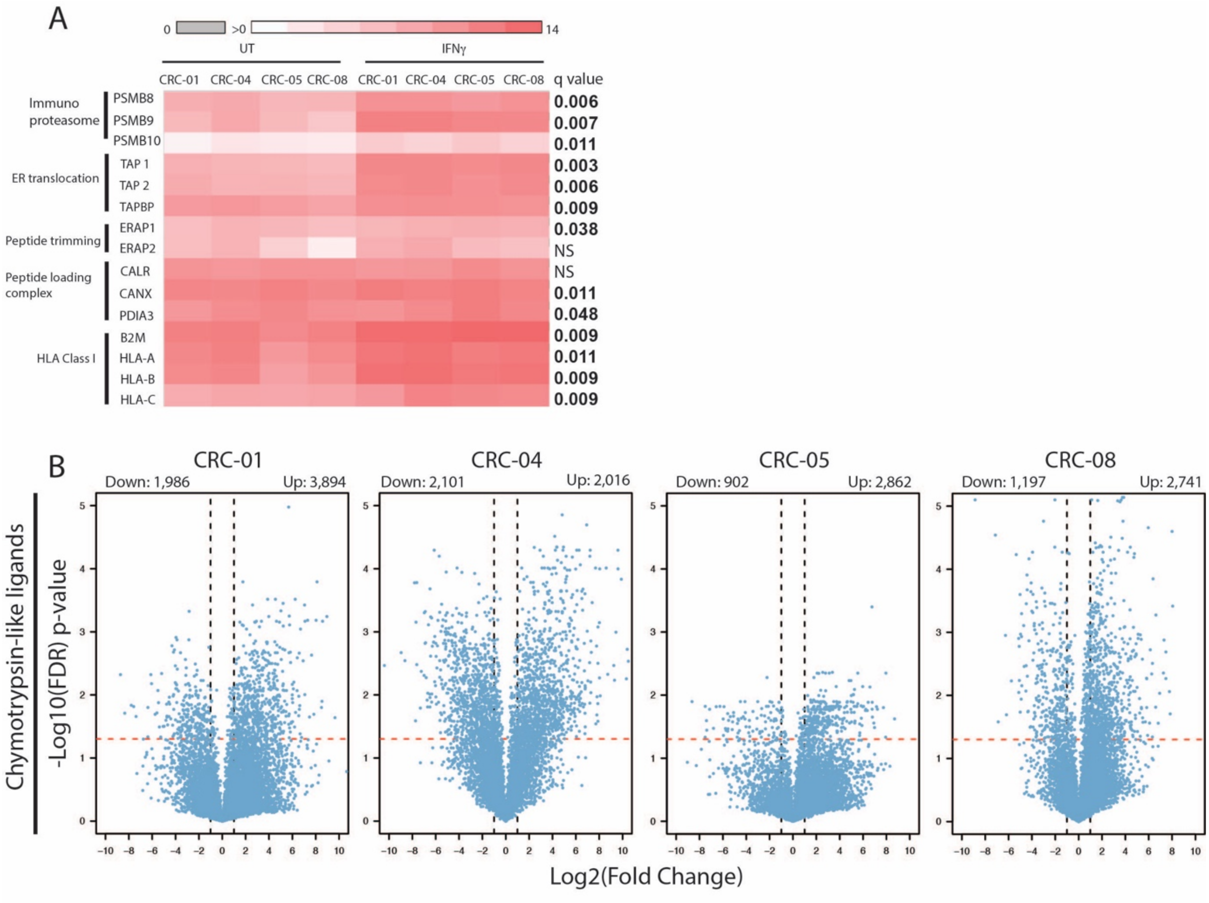

Supplementary Figure 7: Heatmap of changes in key genes for HLA-II peptide presentation induced by IFNγ treatment. Heatmap of key genes involved in HLA-II presentation. Significant difference analysis performed with a paired t-test, followed by FDR analysis set to 5%.

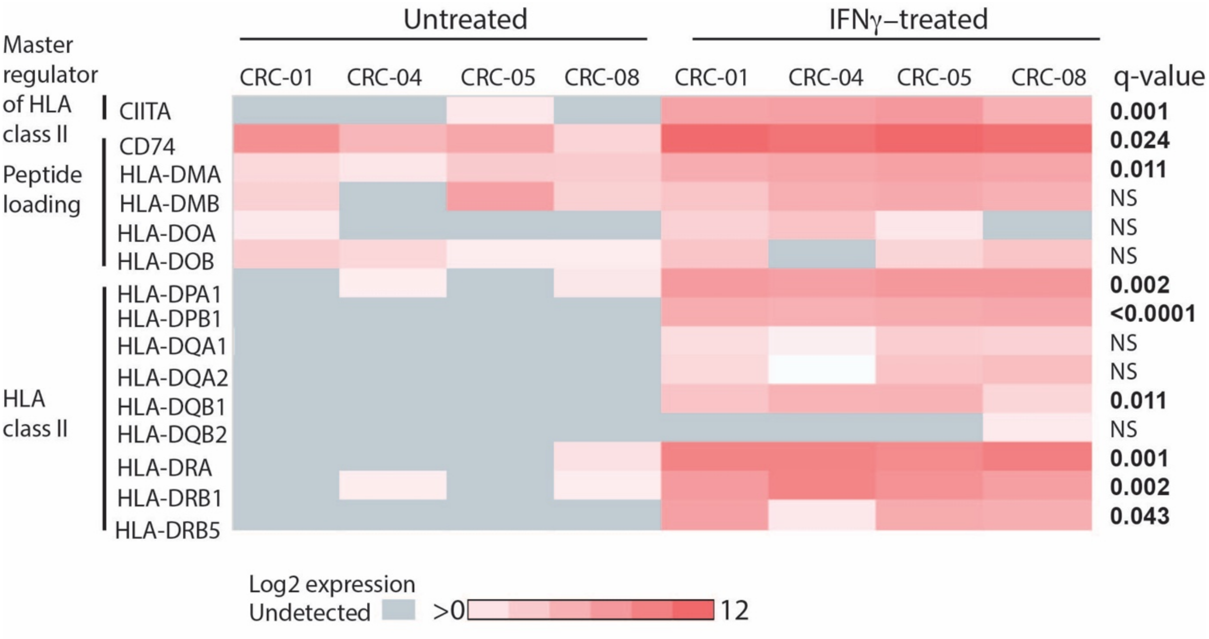

References:

1. Cox J, Mann M. MaxQuant enables high peptide identification rates, individualized p.p.b.-range mass accuracies and proteome-wide protein quantification. Nat Biotechnol. 2008;
2. Racle J, Michaux J, Rockinger GA, Arnaud M, Bobisse S, Chong C, et al. Deep motif deconvolution of HLA-II peptidomes for robust class II epitope predictions. bioRxiv. 2019;
